# Hypocholesterolemic Effects of Nutraceuticals Produced from the Red Microalga *Porphyridium* sp in Rats

**DOI:** 10.3390/nu1020156

**Published:** 2009-11-23

**Authors:** Irit Dvir, Aliza Hannah Stark, Reuven Chayoth, Zecharia Madar, Shoshana Malis Arad

**Affiliations:** 1 Department of Life Sciences, Ben-Gurion University of the Negev, Beer-Sheva 84105, Israel; Email: rchayoth@mail.netvision.net.il; 2 The Robert H. Smith Faculty of Agriculture, Food and Environment, Institute of Biochemistry, Food Science and Nutrition, The Hebrew University of Jerusalem, Rehovot 76100, Israel; Email: stark@agri.huji.ac.il (A.H.S.); madar@agri.huji.ac.il(Z.M.); 3 Department of Biotechnology Engineering, Ben-Gurion University of the Negev, Beer-Sheva 84105, Israel; Email: arad@bgu.ac.il

**Keywords:** red microalgae, nutraceuticals, hypocholesterolemic agents, dietary fiber, foodomics

## Abstract

Red microalgae contain functional sulfated polysaccharides (containing dietary fibers), polyunsaturated fatty acids, zeaxanthin, vitamins, minerals, and proteins. Studies in rat models support the therapeutic properties of algal biomass and isolated polysaccharides. Algal products incorporated into rat diets were found to significantly improve total serum cholesterol, serum triglycerides, hepatic cholesterol levels, HDL/LDL ratios and increased fecal excretion of neutral sterols and bile acids. Morphological and metabolic changes were induced by consumption of algal products. These results suggest that red microalgae can be used as potent hypocholesterolemic agents, and they support the potential use of red microalgae as novel nutraceuticals.

## Abbreviations Used

EPA: eicosapentaenoic acid
HDL/LDL: high density lipoproteins/low density lipoproteins
TG: triglyceride
VLDL-C: very low density lipoprotein- cholesterol

## 1. Introduction

Algae are rich in natural bioactive compounds that may act as antihypertensive, antioxidant, antimicrobial, antiviral, anti-inflammatory, antitumour, anticoagulant and/or hypocholesterolemic agents [1,2,3,4,5,6,7,8,9]. In addition, many species are potential sources of nutraceuticals, since they have high concentrations of vitamins, minerals, phytochemicals, ω-3 fatty acids and various functional polysaccharides [1,2,3,10,11]. Algal products are considered safe for use and have been part of the human diet for centuries. 

Marine algae contain large quantities of soluble and insoluble dietary fibers that differ physicochemically from those of land plants [12,13,14]. Dietary fibers from various marine sources are composed of diverse assortments of polysaccharides that confer on each source a unique chemical composition profile and physical structure [12,13,14]. As a result, different species of marine algae, when eaten by man or animals, will have different physiological and metabolic effects [15]. The mechanisms by which dietary fibers induce physiological changes are related to their viscosities, fermentabilities, water holding capacities, bile acid binding abilities, cation exchange capacities and fecal bulking properties [16]. Animal studies have shown that the consumption of fibers is associated with changes in lipid metabolism, which often include a hypocholesterolemic effect [17,18,19]. It has therefore been hypothesized that the consumption of dietary fibers may decrease the prevalence of diseases associated with low dietary fiber intake, such as obesity, diabetes, heart disease and cancer [20]. 

Studies in our laboratory have shown that the marine red microalga *Porphyridium* sp. constitutes a new source of dietary fibers with hypocholesterolemic potential [3,10]. The cells of this species are encapsulated in a cell wall composed largely of a polysaccharide (about 50‑70% of the biomass) whose external portion dissolves in the culture medium. The cell-wall polysaccharides are heteropolymers (molecular weight of 3–5 × 10^6^ Da) with xylose, glucose and galactose as the primary sugars. The presence of sulfate groups and glucuronic acid confer a negative charge on these molecules [21,22,23]. Like other species of red microalga, *Porphyridium* sp. contains significant amounts of eicosapentaenoic acid [EPA; 20:5 (n-3)] [10,24,25]. 

The consumption of red microalgae has been shown to induce physiological effects, including marked changes in rat intestinal morphology [3]. Significant increases in small intestine and colon length have been documented. Jejunum mucosa and muscularis cross-sectional areas were enlarged and hypertrophy of the muscularis layer was measured following polysaccharide feeding. Furthermore, addition of algal biomass to the diet significantly reduced intestinal transit time [3]. Work in chickens showed that both 5 and 10% levels of algal biomass in the diet were effective in lowering cholesterol levels, and a trend to lower levels of cholesterol in eggs was observed [26]. 

In the present study, we investigated the hypocholesterolaemic effects of algal biomass and of the isolated algal polysaccharide (AP) of *Porphyridium* sp. in hypercholesterolemic rats and sought explanations for the observed mechanisms of action of these effects. 

## 2. Materials and Methods

*Algae and Culture Conditions.* The unicellular red alga *Porphyridium* sp. (UTEX 637) was obtained from the culture collection of the University of Texas, Austin, TX, USA. The algae were cultivated outdoors for 21 days in artificial seawater [27] in polyethylene sleeves [28].

*Biomass Production.* Algal cultures were grown for 14 days. The entire culture was centrifuged at 17,000 × g for 20 min (Cepa Z-41, Carl Parberg, Lahr/Schwarzwald, Germany) and the pellet collected. In order to remove salts, the pellet was washed (deionized water, pH 4). This procedure was repeated and the pellet obtained was then lyophilized and stored in a desiccator until the start of the experiment (up to 3 months). The dry algal cells were designated biomass.

*Algal Polysaccharide (AP) Preparation.* The growth medium was centrifuged and the transparent supernatant containing soluble polysaccharides was collected. This was followed by cross flow filtration (stainless steel equipment, Bio Nes Ltd; filters, A/G Company Ltd) to remove salts. The concentrated algal polysaccharide was frozen (−20 °C), lyophilized and stored in a desiccator until the start of the experiment (up to three months).

*Dietary Fiber Sources and Analyses.* Four sources of dietary fiber were used: (1) cellulose (Solka-folc, James River Corp., Hackensack, NJ, USA); (2) pure citrus pectin (methoxy concentration 10 g·100 g^-1^; Sigma, St. Louis, MO, USA); (3) *Porphyridium* sp. cells (biomass); and (4) algal polysaccharide (AP) of Porphyridium sp. (prepared as described above). Table 1 provides analyses of the dietary fiber sources. Carbohydrate analysis was performed using the phenol-sulfuric method [29]. Fat content was measured by the Kates method [30] and protein content was determined by the Lowry method [31]. Ash content was measured in oven dried samples (70 °C, 24 h). Samples were cooled in a desiccator and weighed. The dried sample was then burned at 600 °C for 24 h. The remaining ash was cooled in a desiccator and weighed. Dietary fiber analysis was carried out by the AOAC standard method [32]. 

*Animals.* Male Sprague-Dawley rats (Harlan, Jerusalem, Israel), each weighing 130–140 g, were housed in individual stainless-steel cages suspended in a controlled environment (22–24 °C and 12 h light/dark), with free access to food and water. Animals were kept according to the guidelines set forth by the Animal Care Committee of Ben-Gurion University of the Negev.

*Diets*.The composition and dietary fiber content of the four different cholesterol-rich diets fed to the rats is presented in Table 1 and Table 2. The algal biomass contained 27% insoluble and 8.5% soluble dietary fibers on a dry weight basis while the AP comprised 37% soluble and 8% insoluble dietary fibers. 

**Table 1:** Composition of the algal polysaccharide (AP), biomass, pectin and cellulose fiber sources.

Ingredient	AP (% dry weight)	Biomass ( % dry weight)	Pectin ( % dry weight)	Cellulose ( % dry weight)
Soluble fibers	37	8.5	89	-
Insoluble fibers	8	27	-	98
**Total dietary fibers**	**45**	**35.5**	**89**	**98**
Carbohydrate	26.5	27	-	-
Protein	15	18	7	1.9
Lipids	2.5	6	-	-
Ash	11	13.5	4	0.1
Total dietary fibers in diet ( %)	6.5	6.5	6.5	2

**Table 2:** Composition of experimental diets.

Ingredient	AP (g·kg^-1^)	Biomass (g·kg^-1^)	Pectin (g·kg^-1^)	Control (g·kg^-1^)
Casein	178	171	196	200
Sucrose	232	232	232	232
Corn starch	325	290	374	420
Corn oil	67	59	70	70
Fiber source	140	190	70	20
Vitamin mix*	10	10	10	10
Salt mix*	35	35	35	35
DL-methionine	3	3	3	3
Cholesterol	10	10	10	10

*Vitamin Mix and Mineral Mix – Standard AIN recommendations. Protein and lipid content was matched for all diets.

*Experimental Procedures.* Twenty-four rats were divided into four groups such that the mean weights of each group were similar. Each group was fed one of the four cholesterol-rich diets (Table 2) for a 15-day period. Every two to three days the animals and feces were weighed, and food intake was assessed. On each of the last four days of the experiment, feces were collected, weighed and frozen at −20 °C. The samples were then dried by lyophilisation and stored in a desiccator for later analysis. On day 14 of the experiment, food was withheld from the rats overnight (12–14 h), and the following morning animals were anesthetized with chloral hydrate 100 g·L^-1^. Blood samples were taken from the heart and placed in tubes with heparin. Samples were then centrifuged for 10 min at 1,000 × g, and plasma was frozen at −20 °C until analysis. The livers were removed, weighed and stored at −20 °C for further analysis. 

*Determination of Plasma Lipids.* Total plasma cholesterol and lipoprotein contents were measured by enzymatic colorimetric methods [33,34]. Plasma triglyceride (TG) levels were determined by the method of Fossati and Prencipe [35]. 

*Fecal and Hepatic Neutral Sterol Analysis*. Neutral sterols were determined by the method of Searcy and Bergquest [36], with the following modifications. Dried fecal or liver samples (50 mg) were saponified with 3 mL of 10% ethanolic KOH for 1 h at 65 °C and then cooled to room temperature. Neutral lipids were extracted as follows: 3 mL of water and 5 mL of petroleum ether (b.p. 40–60 °C) were added to each sample, the contents were mixed for 30 s, and the solvent was removed. The second and third extractions were carried out with 5 mL and 3 mL, respectively, of petroleum ether, and the three extracts were pooled for each sample. The samples were then dried under nitrogen, and 3 mL of acetic acid saturated with FeSO_4_ and 1 ml H_2_SO_4_ were added. Absorbance was read at 490 nm to determine steroid concentrations. A standard curve was prepared with increasing concentrations of cholesterol.

*Fecal Bile Acid Analysis.* Dried fecal matter (100 mg) was extracted overnight with 10 mL of chloroform-methanol [37]. Two milliliters of KCl, 3.7 g·L^-1^, were then added, and the sample was centrifuged at 1,500 × g for 10 min. The upper layer was evaporated to dryness, and the residue was then dissolved in 1 ml of 50% methanol. Total bile acids were determined by enzymatic analysis using a modification of the method of Sheltawy and Losowsky [38], as follows: to 100 μL of sample containing 2 mmol·L^−1^NAD, in phosphate buffer, pH 10.5, were added 3α-hydroxysteroid dehydrogenase (0.1 IU) in a final volume of 1.5 mL. After 30 min at room temperature, the absorbance was read at 340 nm to determine the NADH formed. 

*Statistical Analysis.* Data were expressed as means and standard error. Group means were compared by one-way analysis of variance (ANOVA). Means were considered significantly different at P ≤ 0.05, as determined by Fisher’s projected least significant difference method. 

## 3. Results

**Figure 1:**
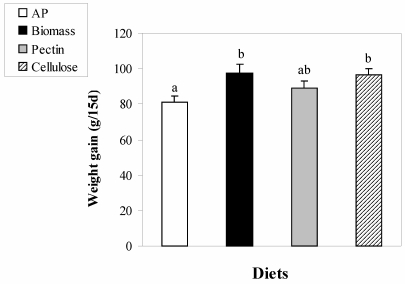
The effect of feeding a hypercholesterolemic diet on weight gain in control rats and rats fed either red microalgal biomass or algal polysaccharide (AP) for 15 days. The isolated fractions contained either soluble polysaccharides found in the growth medium of the algae (AP) or isolated cells (Biomass). Mean values with no common letters were significantly different, p < 0.05. Bars represent SE.

*Body Weight Gain.* Despite similar food intake levels of 6–8 grams per day per rat, significantly lower weight gain (p < 0.03) was observed in AP fed rats (81.3 g) than in the rats fed the control, biomass or pectin diets (97.6 g, 89.1 g, and 96.6 g, respectively, Figure 1). 

*Plasma Lipids and Lipoproteins.* Plasma parameters are presented in Table 3. Cholesterol levels were significantly lower (22–34%; p < 0.001) in rats fed the biomass or AP diets than in control or pectin-fed rats. Similarly, plasma TG and very-low-density lipoprotein (VLDL-C) levels were also lower (by 12–39%) in animals fed AP or biomass. Low-density lipoprotein (LDL) levels were significantly decreased (by 32–53%; p < 0.005) in the polysaccharide-fed rats when compared to rats fed the pectin or control diets. The ratio of high- to low-density lipoproteins (HDL/LDL) was higher (by 31–60%; p < 0.001) in the polysaccharide and biomass groups vs. the pectin and control diets. 

**Table 3:** Plasma lipid levels in rats fed with algal biomass or algal polysaccharide (AP) compared to controls for 15 days (n = 6). Values in columns with different letters differ significantly, p < 0.05.

Study group	Plasma cholesterol (mg/dL)		Plasma triglycerides (mg /dL)		VLDL-C (mg/dL)		HDL/LDL	
	Mean	SE	Mean	SE	Mean	SE	Mean	SE
Polysaccharide	103.17	5.28 ^a^	23.17	1.52 ^a^	46.7	2.9 ^a^	0.77	0.12 ^a^
Biomass	95.67	4.62 ^a^	26.67	1.71 ^a^^b^	53.3	3.4^ab^	0.83	0.07 ^b^
Pectin	132.50	8.62^b^	38.00	2.31 ^c^	76.0	4.6^c^	0.53	0.08 ^bc^
Control	144.00	12.12 ^b^	30.17	3.06 ^b^	60.3	6.1^b^	0.33	0.05 ^c^

*Fecal Dry Weight.* Fecal dry weight was greater in the polysaccharide-fed rats (3.17 g·day^−1^; p < 0.0001) than in the pectin and control groups (0.93 and 1.07 g·day^−1^, respectively). Fecal dry weight was also higher (2.46 g·day^−1^) in the biomass-fed animals (Table 4).

*Fecal Excretion of Neutral Sterols and Bile Acid.* The amount of neutral sterols in the feces was dramatically enhanced in rats fed algal polysaccharide (1.93 mg·day^−1^; p < 0.02) in comparison to the other three treatments (1.19, 0.82 and 0.59 mg·day^-1^ for the biomass, pectin and control groups, respectively) (Table 4). The fecal bile acid content was lowest in the polysaccharide-fed animals (0.33 mg·day^−1^), higher in the pectin and control groups (0.59 mg·day^−1^ and 0.61 mg·day^−1^, respectively) and significantly higher (p < 0.0001) in the biomass group (1.31 mg·day^−1^). Bile acid excretion was more than twofold higher in the biomass-fed rats than in the control animals. 

**Table 4:** Fecal parameters for rats fed experimental diets for 15 days (n = 6). Values in columns with different letters differ significantly, p < 0.05.

Study group	Fecal dry weight (g·day^-1^)		Fecal neutral sterol (g·day^-1^)		Fecal bile acids (g·day^-1^)	
	Mean	SE	Mean	SE	Mean	SE
Polysaccharide	3.17	0.08 ^a^	1.93	0.03 ^a^	0.33	0.07 ^a^
Biomass	2.46	0.08 ^b^	1.19	0.03 ^b^	1.31	0.18 ^b^
Pectin	0.93	0.06 ^c^	0.82	0.04 ^bc^	0.59	0.07 ^a^
Control	1.07	0.05 ^c^	0.59	0.03 ^c^	0.61	0.08 ^a^

*Liver Weight and Hepatic Cholesterol.* The weights of the livers removed from rats fed the biomass and pectin diets (37.76 and 38.08 mg·g body weight^−1^, respectively) were significantly lower (p < 0.0003) than the weights of the control livers (42.86 mg·g body weight^-1^) (Table 5). The lowest liver weight was found in the polysaccharide-fed rats (35.39 mg·g body weight^-1^). Hepatic cholesterol was significantly higher in the control rats (3.89 mg·g liver^−1^; p < 0.0001) than in the polysaccharide-, pectin- and biomass-fed rats (1.89, 1.81 and 2.14 mg·g liver^−1^, respectively).

**Table 5:** Hepatic data for rats (n = 6) fed experimental diets for 15 days. Values in columns with different letters differ significantly, p < 0.05.

Study group	Liver weight (g)		Liver weight (mg·g body weight^-1^)		Hepatic cholesterol (mg g^-1^)	
	Mean	SE	Mean	SE	Mean	SE
Polysaccharide	7.65	0.28 ^a^	35.39	0.81 ^a^	1.89	0.15 ^a^
Biomass	8.90	0.35 ^b^	37.76	1.28 ^a^	2.14	0.05 ^a^
Pectin	8.69	0.37 ^ab^	38.08	0.88 ^a^	1.81	0.22 ^a^
Control	10.24	0.40 ^c^	42.86	0.85 ^b^	3.89	0.5 ^b^

## 4. Discussion

This study examined the therapeutic effects of biomass or algal polysaccharides from the red microalga *Porphyridium* sp. on the lipid metabolism of rats fed diets rich in cholesterol. A significant hypocholesterolemic effect was observed accompanied by a decrease in the accumulation of hepatic cholesterol (Table 5) and by lowered plasma TG and VLDL-C levels (Table 3). These findings are in keeping with a number of recent studies that document the cholesterol- and lipid-lowering ability of algae [18,19,39,40] and that demonstrate the potential health benefits of incorporating algae or algal byproducts into the diet. Indeed, in Asia, seaweeds are traditionally consumed as sea vegetables, but in Western countries they are used mainly as gelling or thickening agents and not as part of the daily diet [11].

Soluble dietary fibers, such as pectin, are known to have hypocholesterolemic effects and are therefore considered important factors in reducing the risk of coronary heart disease [41]. In this study, algal products were as effective as, or more effective than, pectin in lowering plasma cholesterol levels and preventing the accumulation of cholesterol in the liver. Numerous mechanisms have been proposed to account for the cholesterol-lowering action of soluble dietary fibers. One possible explanation is that they increase the viscosity of the intestinal contents, thereby interfering with nutrient absorption and micelle formation, which, in turn, decreases lipid absorption from the intestine [42]. It has also been suggested that soluble fibers act by disrupting the enterohepatic circulation of bile acids, leading to increased bile acid excretion and a subsequent decrease in plasma cholesterol levels [43,44].

Liver weight and hepatic cholesterol accumulation was significantly lower in animals that consumed pectin, AP or biomass. This is of physiological importance as fatty liver is a risk factor associated with the development of chronic liver disease [45]. Natural compounds that have the ability to prevent accumulation of fats in the liver may be used therapeutically as nutraceuticals. 

In this study, consumption of AP resulted in a significant increase in fecal excretion of neutral sterols, while consumption of biomass had a marked effect on fecal excretion of bile acids and a lower but also significant impact on neutral sterol excretion (Table 4). Pectin consumption had a much lower effect on these parameters. Therefore, it is likely that both the biomass and the AG affect lipid metabolism at the level of absorption from the digestive tract. Although both biomass and AP significantly increased fecal weight and possibly interfered with micelle formation, their mechanisms of action may be quite different, due to their unique physicochemical properties (viscosity, solubility, electrical charge, etc.) [22,23,46,47,48] and to the fact that biomass contains primarily insoluble dietary fibers, whereas the majority of fibers in the AP are water soluble. The AP appeared to be more effective in enhancing the secretion of neutral sterols, while the biomass interfered with the enterohepatic circulation of bile acids. Consumption of both biomass and AP resulted in a depletion of total cholesterol pools, which was reflected in lowered hepatic and plasma cholesterol levels. These results corroborate the results of previous studies from our laboratory [3]. In earlier studies, rats fed algal biomass had elevated plasma cholecystokinin (CCK) levels. This may indicate an increased need for bile acid secretion via the gall bladder in order to enhance cholesterol absorption from the digestive tract to compensate for the hypocholesterolemic effect of the algal product. Similarly, HMG-CoA reductase, a key enzyme in endogenous cholesterol production, was found at increased levels in animals fed soluble fibers to lower cholesterol [49]. Feeding AP at levels of 5% or 10% to hypercholesterolemic rats enhanced HMG-CoA reductase in comparison to control animals (unpublished data). 

It is known that fiber-rich diets can inhibit weight gain in rats [50,51]. In our study, the lowest weight gain and a high dry fecal weight (almost three times higher than that of control animals) were observed in AG-fed rats. These findings may be due to the high viscosity of the intestinal contents following food consumption. Indeed, we have previously shown that the viscosity of the cecal contents of AP-fed rats was much higher than that of control animals or pectin-fed rats [3]. Highly viscous chyme may impede nutrient absorption (including cholesterol absorption) along the length of the intestines, leading to lower weight gain [51]. Biomass feeding did not affect weight gain. Similarly, in chickens, the addition of 5% or 10% biomass to the diet did not influence weight gain [26]. 

In the current study, AP (rich in soluble-fiber) significantly reduced plasma TGs and VLDL-Cs. These findings are in keeping with the generally accepted perception that plasma TG levels are lowered by the consumption of soluble dietary fibers [52] and with findings that certain sources of insoluble dietary fibers can lower TG levels, even though most insoluble dietary fibers have little or no impact on lipid metabolism [53,54]. It is also possible that the high EPA content of the biomass, in addition to the fiber content, contributed to the trend of lowered TG levels. 

Other components present in algae may also play a role in the hypocholesterolemic and triglyceride lowering effects observed in this study. Although little information is available, recent studies suggest that pigments such as *C-phycocyanin* present in algae are bioactive compounds and may affect lipid metabolism [55]. 

In conclusion, our work with red microalgae in animal models provides convincing evidence that supports the ability of both biomass and isolated algal polysaccharides (AP) to lower cholesterol levels and positively affect lipid metabolism. Increased fecal bile acid and cholesterol excretion appear to be important mechanisms of action responsible for the overall physiological impact of red microalgae consumption. These results add to the growing body of evidence that the consumption of algal products have health benefits, and as such, algal products can be classified as nutraceuticals.
